# Challenges and perspectives in use of artificial intelligence to support treatment recommendations in clinical oncology

**DOI:** 10.1002/cam4.7398

**Published:** 2024-06-24

**Authors:** Gregor Duwe, Dominique Mercier, Crispin Wiesmann, Verena Kauth, Kerstin Moench, Markus Junker, Christopher C. M. Neumann, Axel Haferkamp, Andreas Dengel, Thomas Höfner

**Affiliations:** ^1^ Department of Urology and Pediatric Urology University Medical Center, Johannes Gutenberg University Mainz Germany; ^2^ Research Unit Smart Data and Knowledge Services German Research Center for Artificial Intelligence Kaiserslautern Germany; ^3^ Department of Hematology, Oncology and Tumor Immunology Charité‐Universitätsmedizin Berlin, Freie Universität Berlin, Humboldt‐Universität zu Berlin Berlin Germany; ^4^ Department of Urology, Ordensklinikum Linz Elisabethinen Linz Austria

**Keywords:** artificial intelligence, clinical oncology, genitourinary cancer, multidisciplinary cancer conferences, treatment recommendation

## Abstract

Artificial intelligence (AI) promises to be the next revolutionary step in modern society. Yet, its role in all fields of industry and science need to be determined. One very promising field is represented by AI‐based decision‐making tools in clinical oncology leading to more comprehensive, personalized therapy approaches. In this review, the authors provide an overview on all relevant technical applications of AI in oncology, which are required to understand the future challenges and realistic perspectives for decision‐making tools. In recent years, various applications of AI in medicine have been developed focusing on the analysis of radiological and pathological images. AI applications encompass large amounts of complex data supporting clinical decision‐making and reducing errors by objectively quantifying all aspects of the data collected. In clinical oncology, almost all patients receive a treatment recommendation in a multidisciplinary cancer conference at the beginning and during their treatment periods. These highly complex decisions are based on a large amount of information (of the patients and of the various treatment options), which need to be analyzed and correctly classified in a short time. In this review, the authors describe the technical and medical requirements of AI to address these scientific challenges in a multidisciplinary manner. Major challenges in the use of AI in oncology and decision‐making tools are data security, data representation, and explainability of AI‐based outcome predictions, in particular for decision‐making processes in multidisciplinary cancer conferences. Finally, limitations and potential solutions are described and compared for current and future research attempts.

## Graphical abstract

Artificial intelligence (AI) promises to be the next revolutionary step in modern society. Major challenges in the use of AI in clinical oncology and decision‐making tools are data security, data representation, and explainability of AI‐based outcome predictions, in particular for decision‐making processes in multidisciplinary cancer conferences. In this review, the authors provide an overview on all relevant technical applications of AI in clinical oncology, which are required to understand the future challenges and realistic perspectives for decision‐making tools.

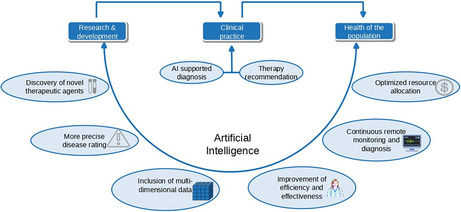

## 1 STATE OF RESEARCH AND POTENTIAL USE OF ARTIFICIAL INTELLIGENCE IN TREATMENT RECOMMENDATION IN CLINICAL ONCOLOGY

### 1.1 Treatment recommendations in multidisciplinary cancer conferences

The field of clinical oncology has become more and more complex over the last decades. Recommending the best available treatment for patients with oncological diseases is a central and complex task. This is commonly conducted in multidisciplinary cancer conferences (MCCs). In these conferences, physicians from various different backgrounds sit together to make comprehensive and complex therapy decisions. The ever more emerging complexity in clinical oncology has made these decisions more difficult as regular updates change first‐, second‐ or third‐line treatments all across tumor entities.^1^, ^2^ For this purpose, national and international medical guidelines offer regularly updated references of medical knowledge for every subspecialty and most common diseases, in order to provide evidence‐based medicine.^3^, ^4^ Medical guidelines represent the basis for treatment decisions made in MCC or by individual physicians.^5^, ^6^, ^7^, ^8^

**FIGURE 1:**
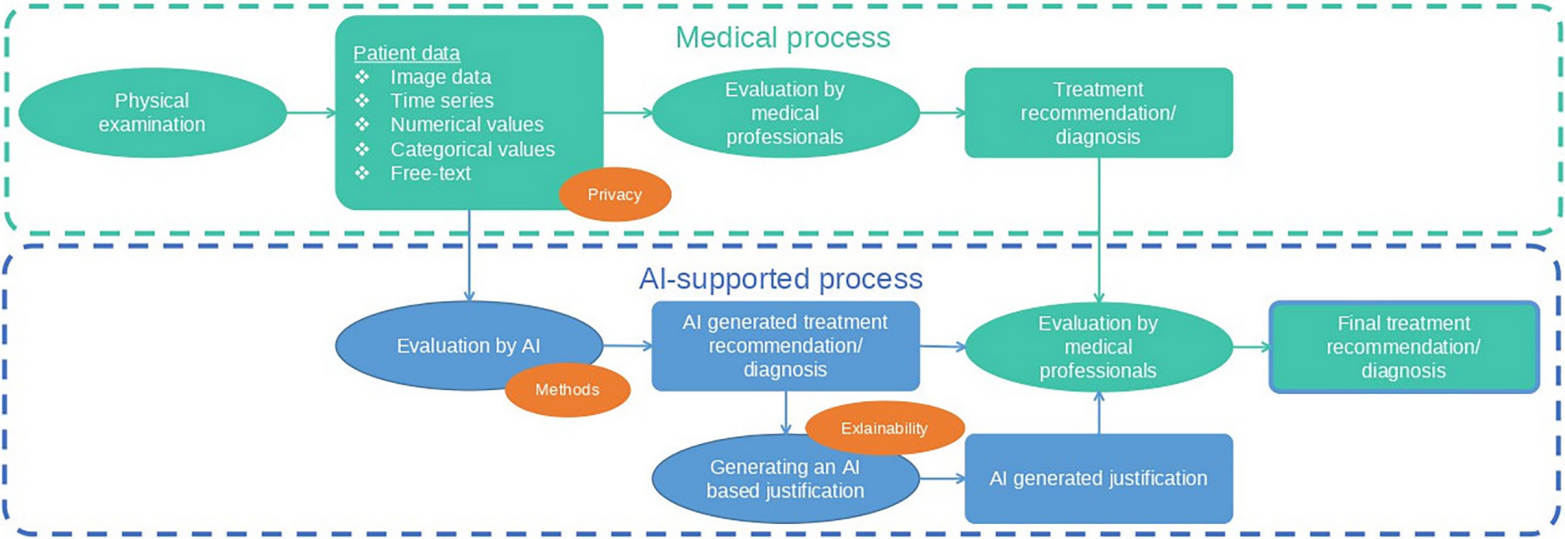
Extension of the medical process by an AI‐supported process, which enables medical staff to support diagnosis and treatment recommendation. This shows an example of a process in which the medical process of generating a treatment recommendation is supported by an AI process. The basic idea is to perform validation after the process, which minimizes the risk of error in the recommendation. First, investigations are performed to collect patient data. This can be any type of relevant data. Next, evaluation is performed by medical professionals and a recommendation is made. The patient data would be evaluated using an AI while maintaining privacy. The AI makes a recommendation, which is presented to the healthcare professional‐ Ideally, an explanation should be generated by the AI that allows the recommendation proposal to be understood. After the evaluation by the specialist, a final recommendation can be made by the medical professionals, which might include suggestions or be supported by the AI‐generated recommendation.

Artificial intelligence (AI) has the potential to reduce this complexity for physicians while increasing the level of evidence‐based treatment recommendations.^9^ We assume it will be possible over the next decade to support physicians in their daily treatment recommendations by generating an objective, evidence‐based AI‐generated treatment recommendation (Figure 1).^10^, ^11^ For instance, there is already the possibility to generate treatment options by using large language models, but their quality remains questionable.^12^ Therefore, it is important that physicians obtain a medical and technical understanding of how AI treatment decision support systems (could) work, how to interpret these results and to understand the current limitations of this new, groundbreaking technology. Consequently, this review aims to give a broad overview for physicians on these computer science‐specific aspects as well as an overview on current applications, future possibilities, and hurdles that need to be overcome. We want physicians to be able to independently understand the technical principles in order to critically scrutinize them before applications are implemented in their everyday lives in the future.

### 1.2 Predicting the clinical success of treatments in clinical oncology

The decision for the right therapy for any cancer disease is always accompanied by the predicted success of a therapy. AI applications are already being used as predictive therapy platforms.^13^ Previous studies using AI to predict treatment response differ in two major outcomes: first, which treatment is being predicted, and second, which source of information is being used to train the AI for the prediction. For both goals, radiological and pathological images as well as clinical parameters can be used as data sources. For example, there are two approaches of AI development based on analyzing histological images. In the first approach, the AI is trained to recognize already known indicators in histological images.^14^, ^15^ This approach is considered less complex because it requires only a small cohort of training images and allows faster prediction based on known indicators. In the second approach, the AI is trained with a much larger cohort of patients, their histological images, and additionally, clinical information of their treatment courses. After the deployment phase, both approaches use the input provided by the doctors directly and present them the results. From user perspective, these systems can be used without any adjustments which provides an “end‐to‐end workflow” as the methodology is a black box for the end‐user. It offers the chance of predicting treatment success in patients for whom this would not have been possible using already known indicators.^14^, ^15^ Currently, only few studies on this approach exist as this requires large datasets to train the AI,^14^ for example, by predicting the success of immunotherapy in non‐small cell lung cancer.^14^, ^16^ In contrast, there are various clinical studies on the first approach, most commonly of histological biomarker for immunohistochemical and genetic analysis.^17^, ^18^, ^19^, ^20^

Until now, radiology offers the broadest range of applications for AI in medicine and oncology, in particular through radiomics.^21^ Radiomics represents a method for the quantitative description of medical images. For example, it is already possible that an AI trained on diffusion‐weighted magnetic resonance images before and after (neoadjuvant) chemotherapy can predict response to therapy in terms of tumor shrinkage after chemotherapy.^22^, ^23^, ^24^ By such approaches, it would be possible to modify or stop chemotherapy in a more individual manner based on treatment success and adverse events.^25^, ^26^, ^27^, ^28^, ^29^, ^30^, ^31^ Recently, scientific approaches of AI were reported in tumor genomics. Here, AI approaches were able to arrange tumor samples based on their RNA and tumor characteristics in a multidimensional space^32^ as well as to predict response to chemotherapy based on tumor RNA.^33^

Moreover, AI applications can be used to predict the survival of cancer patients, representing key information for any treatment decisions in oncology in order to adapt the treatment regime to achieve the highest possible survival outcome as well as quality of life.^34^, ^35^, ^36^ By using data that can be extracted from most medical records, it has already been possible to train AIs that have improved predictions of overall survival for patients.^37^, ^38^ Moreover, a frequently used factor for AI development are patient‐reported outcome measurements which may lead to decrease of mortality, while increasing sensitivity and specificity of the AI.^34^

### 1.3 Use of artificial intelligence applications in medical treatment recommendation

In the past two decades, only few approaches of computer‐based decision support systems have been implemented.^9^ An early example is “OncoDoc,” which was developed for breast cancer patients.^39^ By using a decision tree model based on medical guidelines, “OncoDoc” recommends a treatment option and explains the decision by showing the individual decision tree. However, a case study of OncoDoc's successor “OncoDoc2” showed that 21.3% of the decisions were incorrect and did not follow the medical guidelines.^40^ Another example is the “DESIRRE” project which was running from 2016 until 2020.^41^ This computer‐based decision support system implements different medical guidelines, patient similarity, and the information's and decisions from previous cases in a rule‐based engine, which then gives different treatment recommendations for breast cancer patients.^42^ Until now, no systematic, reliable results have been published to discuss its potential in clinical practice.^43^

The model “Watson for Oncology” (WFO), developed by IBM (International Business Machines Corporation, USA), represents one of the most relevant models in AI Oncology.^44^, ^45^ The goal of WFO was to extract data from any type of medical record in order to make a treatment recommendation based on the most current evidence.^46^ However, the use of WFO in routine clinical practice revealed various problems. One of the major problems was to extract the correct data from medical records. Therefore, despite the market implementation, WFO was still inferior to a physician in interpreting medical texts.^44^ A major hurdle of WFO was the limiting use of the software outside the United States.^47^ The reason for this was that WFO was primarily developed using US guidelines and patient data from a single hospital (*Memorial Sloan Kettering Cancer Center*, *New York*, USA).^45^ This way, WFO was not accepted in real‐world clinical applications across US states or other countries.^47^, ^48^, ^49^ Another major problem with WFO was the “black box,” meaning that WFO could not justify the decisions it made.^46^, ^50^ Because of this and other problems, IBM discontinued the WFO program on January 7, 2022.

A new approach that includes explainability is the KITTU project.^51^ The major goal of this multidisciplinary research project of physicians and experts in AI development is to support multidisciplinary treatment recommendations for patients with genitourinary cancer by using AI applications (Figure 4). One of the most important aspects in this context is to explain the recommendation of the system. With the help of the KITTU project, evidence‐based treatment recommendation in oncology could be increased to improve the quality of treatment and long‐term survival of patients.

## 2 CURRENT STATUS OF TECHNICAL APPLICATIONS OF ARTIFICIAL INTELLIGENCE DEVELOPMENT IN DECISION‐MAKING TOOLS IN CLINICAL ONCOLOGY

The following chapter provides a dedicated overview of existing machine learning approaches in the medial domain with focus in clinical oncology, including relevant examples of use. Furthermore, it provides detailed information about the key challenges and important technical aspects when establishing machine learning in the specific domain of clinical oncology. The following subsections included on possible AI‐architectures, explainability, and privacy as those are the three most important technical, medical, and ethical fields to consider for future developments of a decision support systems in clinical oncology.

### 2.1 Current state of machine learning in medical applications

**FIGURE 2:**
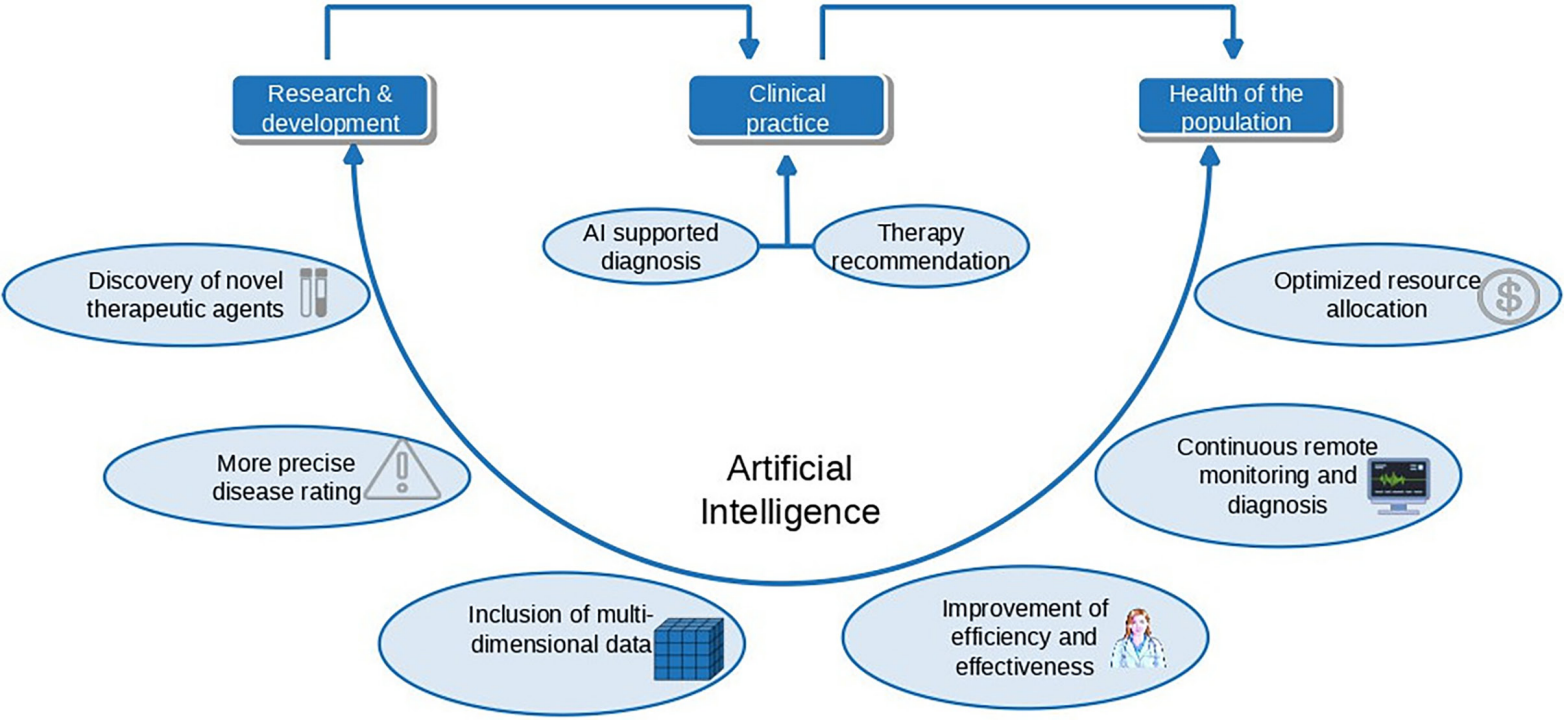
Artificial intelligence represents of the major current research fields in clinical medicine, in particular due to its opportunities in supporting diagnoses and treatment recommendations. This technical support might help to achieve a healthier global population and might have a positive impact on various application fields, such as resource optimization, efficiency, remote monitoring, and others.

**FIGURE 3:**
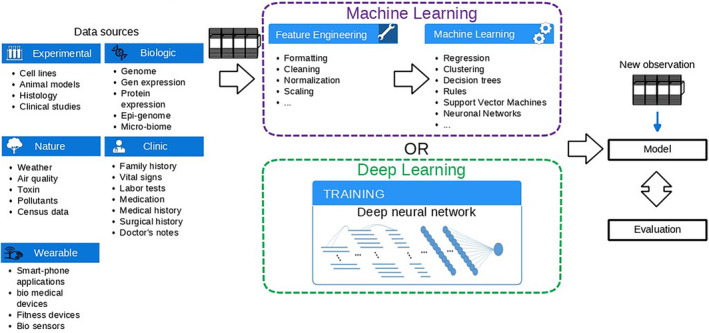
Machine learning can be deployed in various application fields. However, it requires feature engineering. Feature engineering is a complex approach that requires understanding of the data, which can be problematic in some cases. Deep learning can work with almost all kind of data and in addition do not require a costly feature engineering, making it possible to quickly explore the data without prior knowledge.

Since clinical decisions have become more and more complex, machine learning (ML) offers great potential to assist decision‐making. Especially deep structures/deep learning (DL) (Figures 2 and 3) which do not require feature engineering and depend on representation learning to infer data have shown to be successful.^52^ Importantly, these networks do not use pre‐defined rules, but rather work with exemplary data and results. Datasets are used to train computational models, which involve mathematical optimization of neurons. AI models, such as deep neural networks (consisting of many nodes), receive information, process the data accordingly, and make recommendations. Exemplary applications include images of skin lesions to detect skin cancer: during a one‐time training phase, the network is shown images and the corresponding information for each image showing skin cancer or not.^53^ Systems like these process information patterns and is learning to make conclusions on the existence of skin cancer.

Besides the given examples, DL methods are not limited to image data and also work with tabular values, videos, audio, texts, or other sequences.^54^ Potential applications include tasks such as classifications, prediction of values, detection of anomalies, object recognition/labeling, and transformations in the broadest sense. Neural networks require large amounts of data to be trained. Thus, the increasing availability of data and computing power of computer systems led to an increasing interest in AI methods.^55^ DL in medical research is a challenging task for AI experts, because framing the medical problem involves separating two different technical terms for DL. The challenge lies on breaking down complex (human) tasks to a level that is understandable to machine language, but still includes the problem with its complexity. Moreover, data privacy and the largely individual representation of data (e.g., the small amount of training data and its lack of quality) are two key challenges. Therefore, optimal exploitation of datasets represents a major area of research. Furthermore, attempts are made to automatically generate missing data or to synthetically extend the datasets. Another goal of current AI research involves the integration of machine‐readable expert knowledge, for example medical guidelines or clinical studies to push the performance. Lastly, the issue of explainability is one of the major challenges,^56^, ^57^ as it is mandatory for any clinical application to ensure trust and responsibility.

### 2.2 Methods of artificial intelligence development

In recent years, great focus has been placed on “convolutional neural networks” (CNNs), as they can be used in both, image and time series analyses (Table 1). Common issues that can be analyzed with CNNs are medical images including pathological classification of malignant tumors, skin tissues, and other diseases that are visually visible. These networks are able to score predominantly for high accuracy and fast analysis. However, especially in the case of time series, it makes sense to try out other methods in addition to CNNs. For example, “long short‐term memories” (LSTMs)^58^ which were designed to be applied to time series. Another method, originally designed for text processing, is called “Transformer.”^59^ They can handle long time series without losing accuracy. In addition, transformers also provide limited explainability without additional effort by using the attention that can be used to create a kind of heat map for the time series to emphasize on relevant parts.

**TABLE 1:** Illustration how existing problems have been investigated using machine learning methods to give an outlook on the integration of machine learning into the clinical medical context.

References	Target/area of application	Data type	Artifical intelligence	Hybrid	Traditional approaches
(2019)^26^	Predicting the response to immunotherapy in patients with NSCLC	Image (2D)	CNN		
(2021)^18^	Predicting the response to immunotherapy in patients with gastric cancer	Categorical, image (2D)	CNN		
(2019)^17^	Identifying biomarkers to immunotherapy response from histological images of GI cancer	Categorical, image (2D)	CNN		
(2020)^20^	Predicting the response to immunotherapy in patients with colorectal cancer	Categorical, Image (2D)	CNN		
(2021)^33^	Predict the response to chemotherapy in patients with different tumors (5)	Categorical, numeric	CNN, VAE		SVM, XGBoost
(2019)^22^	Predicting the response to chemotherapy in patients with rectal cancer	Image (2D)		Radiomics	SVM
(2020) [43]	Predicting the response to chemotherapy in patients with rectal cancer	Image (2D)	CNN	Radiomics	
(2022)^24^	Predicting the response to chemotherapy in patients with breast cancer before and during therapy	Image (2D)	CNN	Radiomics	
(2022)^25^	Predicting the response of chemotherapy in patients with breast cancer	Image (2D)	CNN, Transformer	Radiomics	
(2021) (^85^)	Predicting textbook outcome of pancreatectomy with sleep and plus data from a wearable	Categorical, numeric			SVM, KNN, Regression
(2021)^36^	Predicting overall survival of patients with gastric cancer	Categorical, image (2D)	CNN		
(2020)^35^	Predicting survival of patients with different (10) tumors	Categorical, image (2D)	CNN		
(2023)^37^	Predicting overall 3‐ and 5‐year survival of patients with metastatic renal cell carcinoma	Categorical, numeric			SVM, KNN, tree‐based, XGBoost, regression

Moreover, it is possible to process texts or tables using AI methods. For this purpose, an important feature is the process of “encoding.” Encoding or embedding of the texts is necessary because an AI has no understanding of words. To enable an AI to process words, they are mapped in a high‐dimensional space. In this process, words that have context are positioned closer together than words without context. Based on encoding, the network can classify texts. Embeddings are not adapted to health terms, therefore it is necessary to train models in such a way that they produce outputs adapted to technical terms.^60^ It is also possible to evaluate tables, by coding values of the table similarly to texts. Regarding medical applications, table analysis enables predictions to be made based on numerous properties which a human being can only take into account all at the same time with great effort. Models, which are used particularly with the analysis of flow texts, are Transformer and LSTMs. Both allow to capture the relationship between the individual words within a text. To give an outlook on the integration of machine learning into the clinical medical context, Table 1 illustrates how existing problems have been investigated using the above‐mentioned methods. In addition to the aforementioned classification and prediction, these methods can also be used for other tasks, for example to mark and track certain features in video or image recordings. Finally, features like completion and generation of data can be used with tabular values and time series^61^ which enables to generate synthetic data that can improve the accuracy of other AI methods.

### 2.3 Explainability of artificial intelligence

To justify the use of AI, it is essential to explain the predictions of these methods. This research field is covered by the so‐called explainable artificial intelligence domain.^56^ It attempts to find an explanation for predictions that is tangible to humans.

One of the most versatile and effective approaches is to look at the relevant data, for example, a heat map that highlights the relevant areas (Figure 5).^62^ To achieve this highlighting, countless different methods have been developed, which can be divided into three categories: gradient‐based, permutation‐based, or replacement‐based. In gradient‐based methods, the backpropagation algorithm utilized during training is used to calculate which information features of the input have led to the output. During the calculation, the network returns a value that represents the influence of each input value. However, these heat maps are not very accurate due to the gradient, resulting into precise details that are not useful for understanding. Permutation‐based approaches modify the input and compute a change in the output. An assumption made here is that modifying relevant input values also results in a change of the output. This approach contains less noise and is easier to interpret. The third category attempts to have the complex AI model mimicked by a simpler explainable model.

There are other ways to explain predictions of AI models, so‐called ante hoc methods. The goal is to modify the AI models in a way that their prediction can be interpreted directly. This usually leads to a decrease in accuracy. A well‐known method is the so‐called “attention,”^63^ which is used by transformers. Here, a heat map of the input is already created during the prediction. In contrast to most other ante hoc explainability methods, transformers show very high performance and are less bound to an architecture. Prototypes are a widely used way to explain the prediction of an AI network. Prototypes are specific salient features/patterns. For example, a specific pattern within an image that is crucial in certain diseases. For a prediction, the AI compares the input data with the prototypes it has learned on its own and explains its decision by highlighting the recognized prototype (salience/pattern). Another possibility for achieving explainability is to use a second network to compress the input in such a way that it shows only the features that are used by the actual network.^64^

### 2.4 Privacy and data security

Neural networks are learning based on a training datasets and store information about the data in this context. It is possible to reconstruct training data to obtain sensitive information. Additionally, it requires prevention to protect the models against external attacks for data extraction. It is important to understand attack methods and their impact on the models. One common attack method is the membership attack,^65^ in which an attempt is made to obtain information about training data from the model by means of targeted queries. In conjunction with this, it is possible to reconstruct the dataset and then train a custom model. With such a model reconstruction,^66^ an attempt is made to train a model of one's own, which copies the original, by making special queries to the private model. Many more approaches exist to steal sensitive information from AI methods. Classical anonymization can only prevent this to a limited extent, since there are also methods to reverse this.

There are different methods that can be used to protect against such attacks. One of the best‐known is “homomorphic encryption,” in which the model and data are encrypted in the following way^67^ that only the parties involved can use them. This makes it possible to protect a model without suffering a loss of performance. Unfortunately, it is not yet possible to apply this approach to larger networks because the additional computational cost is enormous. One option to avoid this is to encrypt the model only after training, thus protecting the model after training but not during the training phase. A widely used alternative is “Differential Privacy.”^68^ Here, data and optimizers of the network are already modified during training so that the resulting model protects the sensitive data. “Noise” is added during training, and security depends on the strength of the noise. This method can be applied to almost all network architectures, but it weakens the accuracy of the models. There is also the option to have a model which learns artificially distributed.^69^ Especially, the distributed processing can be used to aggregate data across multiple institutions to create datasets large enough to train neural networks, however, the sharing involves further security aspects. During the training process, the gradients used for optimization are rounded. In the second step, the average of the computed gradients of these models is used to update the model. The generated average of data information makes the model less sensitive to attacks. The method can be combined with differential privacy models. In generative approaches, synthetic data is generated. A protected generative model uses the sensitive data to generate synthetic data that cannot be distinguished from the original data by an attacker. To do this, the generative models, usually generative adversarial networks (GANs)^70^ or variational autoencoders (VAEs) are trained using differential privacy. The generated data can be used by a model without any protection mechanism.

### 2.5 Usage of medical data: Representation, types, quality, and quantity

In addition to the distinction between imaging, text, video, and tabular data, a more precise division is necessary, especially for tabular data. Data are distinguished in numeric and categorical data. Numerical data can be used directly by AI methods for the most part, while categorical data must be converted into a machine‐readable format. Ordinal data include a relationship between the values, while nominal data has no relation. However, both must be transformed into categories. One approach that transforms categories into numerical values without a cardinal relationship is “one‐hot encoding.” For textual data, it is further required to design a context‐specific vocabulary (Embedding) that maps the words to a numerical representation.

The amount of data is one of the biggest problems regarding any medical projects of AI development. Specifically, AI models are known to handle a larger amount of data than traditional models such as support vector machines,^71^ linear regression,^72^ or decision trees. Furthermore, there is no strict rule of how many samples are required to successfully learn a task, leading to the problem that collecting data results in the risk of having not enough samples or samples with too much variety to establish a working algorithm.

Finally, one of the largest challenges is the preparation of the data, as the representation used by physicians and AI differs largely. This means that in most cases, many efforts are required to first extract and then transform the data into a representation that is suitable for an AI system. Depending on the chosen model, this extraction involves a feature as shown in Figure 3. The data preparation further includes the adjustment of data quality. As the medical process is continuous and improves over time, the data representation changes leading to different representation of similar features, empty data fields, different terms, and some mistakes in the data. All those aspects need to be addressed during the extraction and preparation. Therefore, one need to implement rules, incorporate logical rules, and ontologies to merge terms.

### 2.6 Continuous lifelong learning: Adaption of approaches

One of the biggest challenges is to keep the approaches updated. In the medical domain, there are always new medications or other treatment options that need to be considered by an automated system. If this is not the case, the system works with a snapshot of patient data and does neither improve nor keep the quality at a desired level. One naive approach is to retrain the system from scratch, but this can result in huge resource consumption and requires time. Another approach is to fine‐tune the model using some of the old data and new data. Fine‐tuning is much faster and requires less resources, furthermore, it benefits from the old system that already learned the concepts of the task. However, the term fine‐tuning is mainly used when a network is adapted to a new dataset. Modifying the task without forgetting old relevant information is called continuous lifelong learning. There are several works that review this kind of approaches.^73^, ^74^ Furthermore, scientific attempts showed that it is possible to adapt a network to new data^75^ making it possible to create AI approaches that learn continuously to always produce state‐of‐the‐art results. However, it has to be mentioned that even is such a scenario, it is important to update the AI approaches using novel technology such as better architecture and apply the life‐long learning on these approaches.

## 3 FUTURE PERSPECTIVES IN TECHNICAL APPLICATIONS OF ARTIFICIAL INTELLIGENCE IN CLINICAL ONCOLOGY

### 3.1 Perspectives in usage of artificial intelligence applications in medical treatment recommendation

Data should ideally always be up‐to‐date for the purpose of “AI training.”^76^ Qualitative and quantitative, high‐quality data are an important factor in increasing the evidence of AI. To further increase evidence, it is necessary for an AI to be validated by external validation. The example of WFO showed that this is not granted at all. One of the central problems of WFO was the fact that it had not been trained with representative data.^45^ Therefore, it is of great importance that training data of AI are representative for all patient groups.^76^ The highest possible level of evidence provides patients a high level of safety in treatment. In this context, it is important to know who is legally responsible if an AI in case of potential user mistakes with consequences for the health of patients. Thus, it is mandatory that physicians are well‐trained in the use of AI applications.^77^

### 3.2 Opportunities and limitations

**FIGURE 4:**
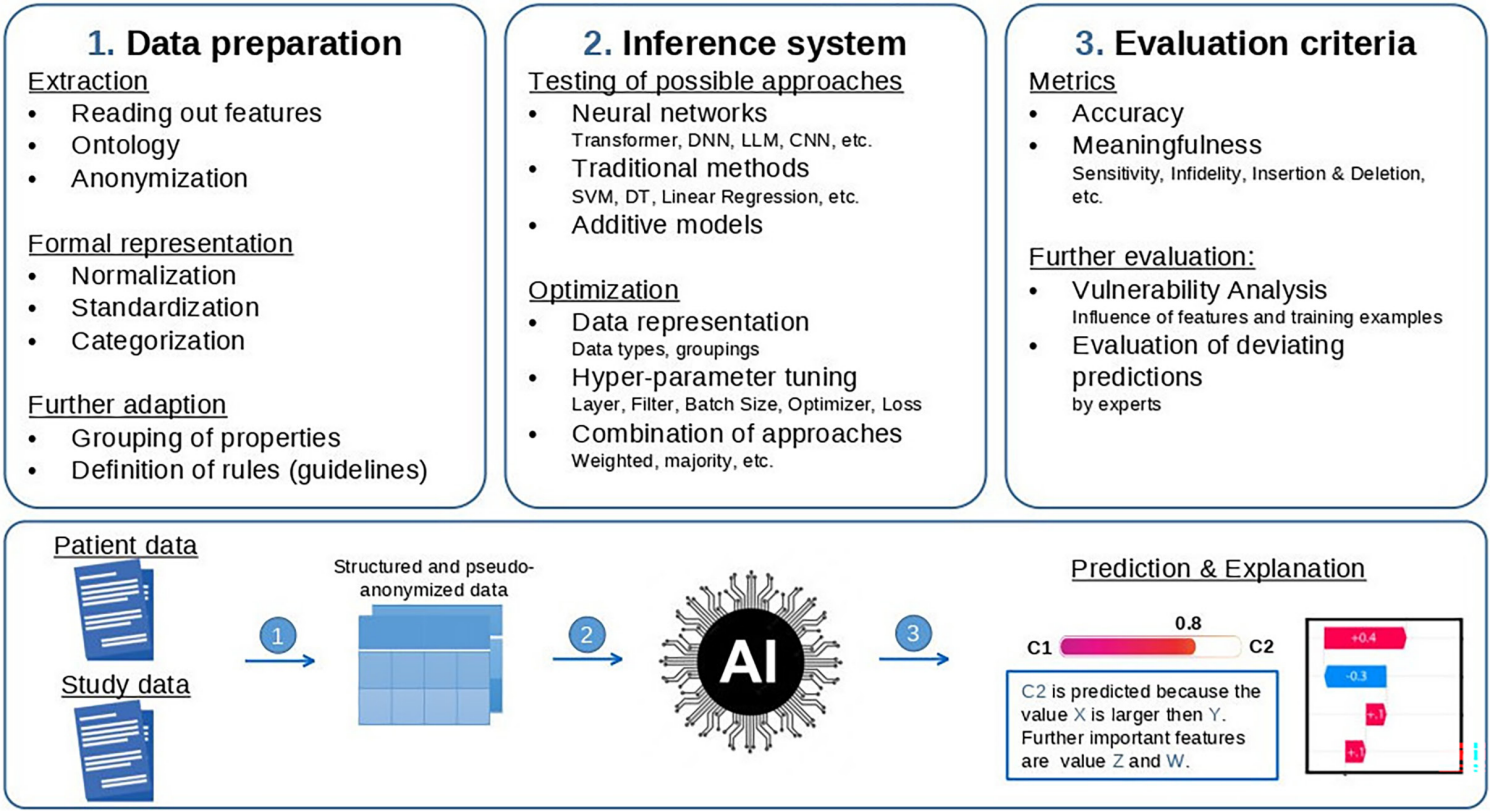
Illustration of different working steps required to develop a trustful system for medical use cases. First (1), a reliable data extraction is required to gather information from different sources and standardize it in a way that is readable for the machine learning. Second (2), an inference system needs to be developed and optimized for the use case. To achieve the best result, multiple approaches need to be tested, optimized and possible combinations need to be evaluated. Finally (3), the accuracy and explainability need to be evaluated including further analysis by experts and a vulnerability analysis to ensure a reliable and trustful system.

A major goal towards the best possible treatment in oncology is personalized medicine.^76^ For this purpose, AI applications allow a maximum high number of data and images of a single patient to be analyzed automatically in a very short time in order to determine the best treatment available^11^, ^78^ (Figure 4). This capability is particularly helpful with complex oncological diseases, which to this date are evaluated and treated by MCCs (Figure 1). Here, AI could help physicians by focusing on the key information from large databases of patients' characteristics and from trial information of available treatments, which would be analyzed by AI applications.^79^ AI could incorporate all existing, relevant medical publications of available treatment regimens into their decision without their own interpretation, which are not (yet) known to a treating physician.^44^, ^47^, ^80^ Furthermore, human errors or physician‐dependent individual variation could be decreased.^81^ By processing data quickly and automatically, an AI would be able to reduce time in various settings during the treatment process in oncology. AI applications could offer a cost‐reducing effect by reducing working time and replace expensive tests and examinations.^17^, ^19^, ^20^

**FIGURE 5:**
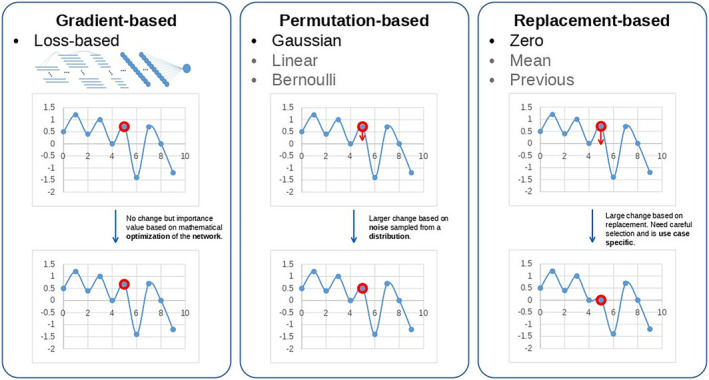
Illustration of different concepts used in attribution methods to produce a “heat map.” Gradient‐based approaches depend on the loss and require a backward pass of the data to mathematically compute the importance value of each point. Permutation‐based approaches only require forward passes and change the input to understand the impact of input changes. Replacement‐based approaches are related to the previous mentioned approaches but are different in the way that they do not perform a small change of the point but rather completely remove the point or set it to a fixed value.

However, we need to be aware of AI's limitations and current problems. A major problem is the “black box” which describes the problem of the missing explainability of decisions by AI.^46^ The ability of an AI to justify its decision is almost mandatory for a clinical implementation. There are already a few, promising AI applications in predicting treatment response that can justify their decisions and are referred to as “white boxes.”^82^, ^83^, ^84^ Finally, we need to consider the immense financial investment and associated financial risk that would be necessary to develop and implement AI in global healthcare systems.^77^

## 4 CONCLUSION

Applications of AI already offer many potential advantages and will be able to offer even more benefits in the future. These applications are only as good as the data is on which they are trained. Thus, it is essential to use data of highest quality and quantity. Both conditions can be significantly achieved through maximum digitization in our healthcare system. Data which will be used to train any AI should be generated using standards that are as uniform as possible.^78^ In future, such a standardization should take place on a global level across different healthcare system in order to improve AI developments and applications globally.^76^ Finally, it will be mandatory to explain any AI‐generated recommendation in order to achieve a successful implementation of any AI applications in clinical practice. Nevertheless, the final decision should always be made by the physicians in a joint decision‐making process with the patients.

## AUTHOR CONTRIBUTIONS

**Gregor Duwe:** Conceptualization (equal); funding acquisition (equal); supervision (equal); validation (equal); visualization (equal); writing – original draft (equal); writing – review and editing (equal). **Dominique Mercier:** Conceptualization (equal); resources (equal); validation (equal); visualization (equal); writing – original draft (equal); writing – review and editing (equal). **Crispin Wiesmann:** Resources (equal); visualization (equal); writing – original draft (equal); writing – review and editing (equal). **Verena Kauth:** Writing – original draft (equal); writing – review and editing (equal). **Kerstin Moench:** Writing – original draft (equal); writing – review and editing (equal). **Markus Junker:** Conceptualization (equal); funding acquisition (equal); validation (equal); writing – review and editing (equal). **Christopher C. M. Neumann:** Supervision (equal); validation (equal); writing – review and editing (equal). **Axel Haferkamp:** Conceptualization (equal); supervision (equal); validation (equal); writing – review and editing (equal). **Andreas Dengel:** Conceptualization (equal); funding acquisition (equal); supervision (equal); validation (equal); writing – review and editing (equal). **Thomas Höfner:** Conceptualization (equal); funding acquisition (equal); supervision (equal); validation (equal); writing – review and editing (equal).

## FUNDING INFORMATION

This work was supported by the German Federal Ministry of Education and Research, grant number: 16SV9053.

## CONFLICT OF INTEREST STATEMENT

The authors declare no conflicts of interest.

## ETHICS STATEMENT

No ethics approval is required as data is freely available online in the public domain.

## Data Availability

Data sharing not applicable to this article as no datasets were generated or analyzed during the current study.
